# Cracking the (tubulin) code of mitosis

**DOI:** 10.18632/oncotarget.5108

**Published:** 2015-08-06

**Authors:** Marin Barisic, Helder Maiato

**Affiliations:** Chromosome Instability & Dynamics Laboratory, Instituto de Biologia Molecular e Celular; Instituto de Investigação e Inovação em Saúde (i3S); Cell Division Unit, Department of Experimental Biology, Faculda de Medicina, Universidade do Porto, Porto, Portugall

**Keywords:** chromosomes, microtubules, detyrosination, motor proteins, mitosis

Mitosis in high eukaryotes is typically initiated by nuclear envelope breakdown (NEB), consequently allowing the dynamic interaction between microtubules and condensed chromosomes. These spatially scattered chromosomes congress to the spindle equator to establish a metaphase plate before being equally distributed to two daughter cells during anaphase. Congression can be achieved by at least two mechanisms, depending on the initial position of each chromosome at NEB. Chromosomes that are initially located between two newly formed spindle poles have a greater chance to become bi-oriented, with each kinetochore from the pair attached to microtubules from opposite poles, and align via microtubule polymerization/depolymerization coupled movement. Alternatively, peripheral chromosomes that are positioned more distantly from this interpolar zone at NEB and are in closer proximity to one of the two spindle poles, are initially captured by lateral attachments between astral microtubules and kinetochores and subsequently transported towards the pole by the microtubule minus-end-directed activity of Dynein at kinetochores. Once the chromosome reaches the pole, the microtubule plus-end-directed kinetochore protein CENP-E overtakes Dynein and drives chromosomes towards the spindle equator [1]. While Dynein-powered chromosome movement towards the pole is easily understandable since all microtubule-minus ends in the spindle terminate in the centrosomal region, how CENP-E guides chromosomes preferentially towards the equator has remained enigmatic. Indeed, the plus-ends of microtubules originated at the spindle poles do not point exclusively towards the spindle equator, and many (astral microtubules) are oriented towards the cell cortex. Thus, a key unanswered question was how CENP-E distinguishes different microtubules to drive peripheral chromosomes exclusively towards the spindle equator.

Recently, we addressed this question by investigating the impact of post-translational modifications (PTMs) of tubulin on CENP-E activity and function. This was based on the hypothesis that the activity of motor proteins is not affected exclusively via its molecular regulation (e.g. by phosphorylation), and might also be achieved indirectly, by modifying the tracks in which they move on (e.g. by post-translational modifications of microtubules). Although this possibility was completely unexplored in mitosis, several studies had reported a correlation between Kinesin-1 activity and tubulin PTMs in neurons [2-4]. As so, we have initially tested whether microtubule acetylation played a significant role in chromosome congression. Predictably, if tubulin acetylation would affect the activity of CENP-E, we should observe a phenotype similar to the depletion or inhibition of the motor itself. However, we could not detect any major defects in chromosome congression after inhibiting alpha-tubulin acetyltransferase 1 (α-TAT1), the enzyme that inserts the acetyl group into microtubules [5].

**Figure 1:**
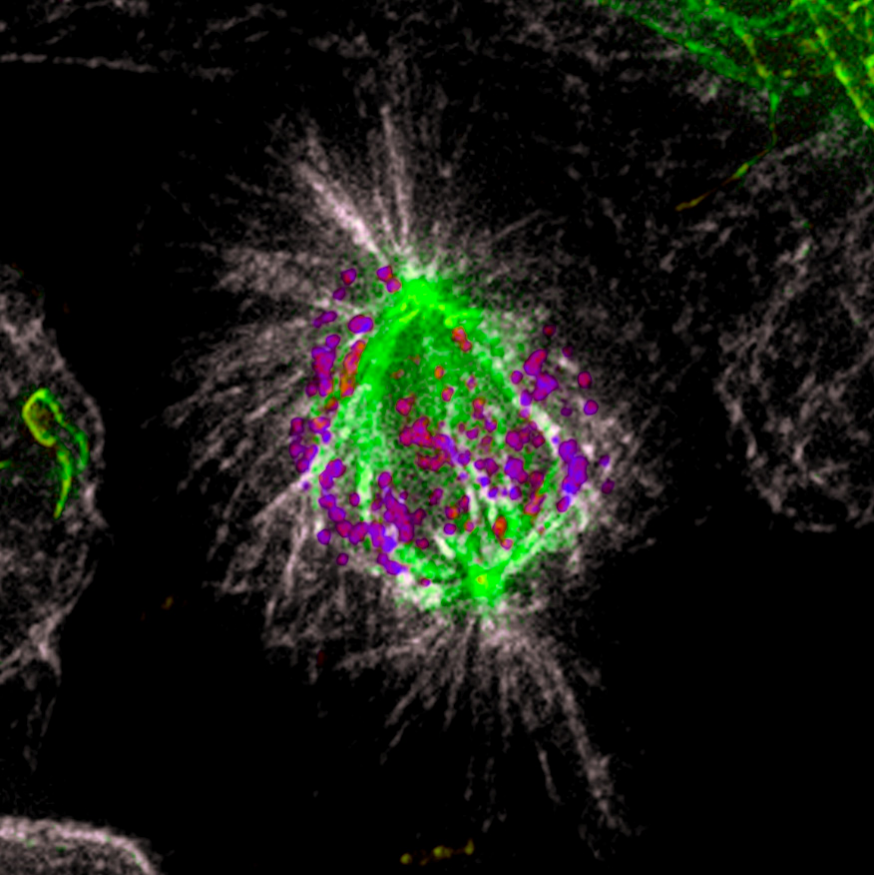
Microtubule detyrosination guides chromosomes to the spindle equator Immunofluorescence of a methanol fixed U2OS cell in mitosis. Total α-tubulin in white; detyrosinated tubulin in green; kinetochores (ACA) in purple. Note that the astral microtubules are not detyrosinated.

Unlike tubulin acetylation, which takes place on the luminal side of microtubules, tubulin detyrosination occurs at the very end of α-tubulin c-terminal, and is located at the outer surface of the microtubule lattice, where it is more likely to interact with motor proteins. In addition, almost three decades ago, it was shown that only a subclass of stable spindle microtubules pointing to the equator is detyrosinated, while highly dynamic astral microtubules remain tyrosinated [6](Figure 1). Thus, microtubule detyrosination became a particularly attractive candidate to restrict CENP-E-mediated motion exclusively towards the spindle equator. To test this hypothesis, we developed two strategies to decrease the levels of microtubule detyrosination in cells. First, we overexpressed tubulin tyrosine ligase (TTL), the enzyme that specifically adds back tyrosine to the α-tubulin c-terminal tail [7]. Additionally, we used the chemical compound Parthenolide to inhibit the yet unidentified tubulin carboxypeptidase (TCP) that removes the c-terminal tyrosine from α-tubulin [7]. Both treatments revealed cellular phenotypes identical to CENP-E depletion/inhibition, consisting of a group of chromosomes (∼15%) [1] that remained “locked” at the spindle poles, and were unable to congress to the metaphase plate for several hours. These results indicated that microtubule detyrosination might be required for CENP-E-mediated chromosome motion from the poles towards the spindle equator, while Dynein minus-end-directed activity keeps those chromosomes “locked” at the poles. To test this we designed the opposite experiment in which we forced detyrosination of all spindle microtubules, including astral microtubules, by RNAi-mediated depletion of TTL. The prediction was that, once all microtubules become detyrosinated, CENP-E remains processive and dominant over Dynein, but it looses the bias towards the metaphase plate, while being able to walk in any direction, including towards the cell cortex along detyrosinated astral microtubules. Indeed, after TTL RNAi cells revealed congression problems, with a group of chromosomes being ejected from the spindle pole towards the cell cortex. Importantly, these movements were dependent on CENP-E, since we were able to significantly rescue this phenotype by inhibiting its motor activity. Finally, we directly tested our hypothesis in an *in vitro* reconstitution assay, examining the motility of recombinant CENP-E on purified tyrosinated vs. detyrosinated microtubules. Both the *in vitro* motility assay and optical trapping experiments revealed that CENP-E dependent transport was strongly enhanced on detyrosinated microtubules, confirming the previously observed cell phenotypes [5].

Overall, these data demonstrate that microtubule detyrosination, as part of the tubulin code, affects the activity of a kinetochore-based motor protein and works as a navigation system for chromosome movements in mitosis. In the future, it will be important to investigate whether the tubulin code impacts the function of other motor proteins required for different aspects behind mitotic spindle architecture and function.
